# Effects of Irradiation on Biology and Mating Behaviour of Wild Males of Brown Marmorated Stink Bug Using a 6 MV Medical Linear Accelerator

**DOI:** 10.3390/insects14050460

**Published:** 2023-05-13

**Authors:** Gerardo Roselli, Gianfranco Anfora, David Maxwell Suckling, Valerio Mazzoni, Valentina Vanoni, Loris Menegotti, Lorenzo Fellin, Marco Valerio Rossi Stacconi, Claudio Ioriatti, Massimo Cristofaro

**Affiliations:** 1 Center Agriculture, Food and Environment (C3A), University of Trento, 38098 San Michele all’Adige, Italy; gerardo.roselli@unitn.it (G.R.);; 2 Technology Transfer Centre, Fondazione Edmund Mach, 38098 San Michele all’Adige, Italy; 3 Biotechnology and Biological Control Agency (BBCA Onlus), 00123 Rome, Italy; 4 Formerly The New Zealand Institute for Plant and Food Research Ltd., Christchurch 8011, New Zealand; 5 Formerly School of Biological Sciences, University of Auckland, Auckland 1072, New Zealand; 6 Research and Innovation Centre, Fondazione Edmund Mach, 38098 San Michele all’Adige, Italy; 7 Azienda Provinciale per i Servizi Sanitari, 38122 Trento, Italy

**Keywords:** X-ray, *Halyomorpha halys*, integrated pest management, insect pest, pentatomids, diapause

## Abstract

**Simple Summary:**

Controlling the brown marmorated stink bug without chemical insecticides is challenging. The sterile insect technique, based on use of irradiated sterile males to reduce fertility of wild females, is a valid method in area-wide pest management. This work complements previous investigations that were carried out by treating newly emerged males at lower irradiation doses. In this study, high irradiation doses (32 and 40 Gy) were applied, using a linear accelerator, to a colony of wild overwintering adults collected in large numbers in the field during the aggregation phase before the winter diapause. A sterility level of 95% was reached with a minimum 32 Gy X-ray irradiation dose and without significant impacts on other physiological parameters, such as fecundity and longevity.

**Abstract:**

The brown marmorated stink bug, *Halyomorpha halys*, is a pentatomid bug of Eastern Asian origin that became an economically relevant pest in the Eurasian and American continents. Management of this species is limited to use of chemical insecticides: an inefficient method due to the strong adaptability of the target pest. The sterile insect technique (SIT) is potentially a valid tactic in the search for nontoxic alternatives. In this work, we investigated the suitability of mass-trapped overwintering males, collected during the aggregation phase before the winter diapause, for their release as competitive sterile males in an SIT programme. Differently from previous studies, irradiation was applied with a linear accelerator device that produced high-energy photons. Following a similar scientific protocol with newly emerged irradiated males, the effects of X-ray irradiation on physiological parameters (longevity, fecundity and fertility) were assessed. In addition, behavioural bioassays were carried out in no-choice conditions to evaluate if irradiation interferes with mating processes. The results are very encouraging; the effects of the irradiation at 32 Gy did not differ from the controls in the longevity or fecundity of the exposed overwintering adults. The hatching rate of the eggs laid by the fertile females that had mated with the irradiated males was less than 5%. The results of behavioural bioassays showed that the irradiation did not cause a significant impact on the quality of the sterile males. More research is warranted to evaluate the mating competitiveness of sterile males in semi-field and field conditions.

## 1. Introduction

The brown marmorated stink bug (BMSB), *Halyomorpha halys* (Stål) (Hemiptera: Pentatomidae), is an invasive phytophagous species native to Eastern Asia [1]. Due to its wide host-plant range, it is considered among the most harmful agricultural pests in Europe and the United States [2,3]. In addition, in the southern hemisphere, regions with moist and tropical, subtropical, Mediterranean and warm-temperate climates are at risk of establishment of BMSBs [4]. Apples, peaches, nectarines, pears, grapes, sweet corn, soybeans and hazelnuts are among the most susceptible cultivated hosts [3]. In addition, this insect can cause nuisance in urban areas by invading buildings as shelters for overwintering and by emitting an unpleasant smell when disturbed [5,6,7]. Management of *H. halys* relies mainly on insecticide use, in particular, pyrethroids and neonicotinoids, in combination with other agronomic and biocontrol strategies [8,9,10]. There is a desire to limit use of broad-spectrum insecticides in favour of alternative, less-hazardous techniques in integrated pest management (IPM). Use of both native and exotic natural biocontrol agents is showing promising mid- and long-term results against BMSBs [11,12]. Another potential control tactic is the Sterile Insect Technique (SIT). By definition, SIT is an environmentally-friendly pest control strategy based on mass rearing, sterilization, and inundative releases of predominantly male sterile insects. It is species-specific control method, and no documented off-target effects have been reported after decades of application [13]. To achieve sterility, mass-reared insects are typically irradiated with gamma rays or X-rays, making them unable to produce fertile offspring. Populations reduce when enough sterile males are mated with wild females, [14]. The Sterile Insect Technique has been succesfully used to control insect pests in many ecosystems. For instance, SIT has protected the horticultural industries from *Ceratitis capitata* (Wiedemann) (Diptera: Tephritidae) in many countries such as Guatemala, Belize, Mexico, and the USA [15]; SIT applied against the tsetse fly *Glossina austeni* (Wiedemann) (Diptera: Glossinidae), made its eradication possible from Unguja Island, Zanzibar where it represented a severe problem for livestock [16]. Finally, SIT is used in various programs to control different species of mosquitoes, which are vectors of pathogens causing serious human diseases such as malaria and dengue [17]. To guarantee a successful outcome of SIT, many assumptions must be met, such as the absence of parthenogenesis, the possibility of mass rearing of the insects, a good knowledge of the insect mating behaviour and reproductive biology, size, and dispersal of natural populations, little to no adult crop damage, and no risks for human health and animals (for instance by transmitting pathogens). In the case of invasive hemipterans, the main obstacle to SIT application as a control method is the unwanted damage to host crops that could be caused by the release of adult pests, despite them being sterile [13]. Nonetheless, the application of SIT is particularly capable of giving successful pest suppression and even eradication, regardless of the challenges, when applied in specific geographic and infrastructural conditions with a closed population such as islands, greenhouses and siloes with particular respect to several conditions [14].

To prepare for a possible application of the SIT against BMSBs, previous studies investigated the effects of irradiation on different phenological stages [18,19,20]. To date, however, the SIT has not yet been included in any control strategy against *H. halys* despite the great potential it offers for some situations. More studies are needed to develop a comprehensive and affordable technique before deployment. A critical problem facing SIT implementation on brown marmorated stink bugs is the impracticability of sustainable mass rearing. It has been proposed that this challenge could be mitigated by rearing, sterilising and releasing populations of previously wild-harvested aggregated overwintering males [19]. 

Overwintering is an important phase for both male and female adults of BMSB; the process is extremely selective since it is characterised by extremely high mortality. For example, Costi et al. [2] observed that in Emilia Romagna (Italy), in outdoor conditions, only 28% of BMSB adults survived overwintering, and only half of them (14%) were capable of reproducing. Moreover, it has been demonstrated that BMSB females overwinter unmated and sexually immature [21], and to reach sexual maturity, they have to go through a period of cold storage followed by a postdiapause period under specific temperature [22] and photoperiod [21] conditions. Our preliminary observations on about 11,000 overwintering adults, collected during autumn of 2018, showed that BMSBs need seven weeks of winter diapause in outdoor conditions, followed by a three-week postdiapause period in controlled conditions before oviposition. While the postdiapause period is essential for females to reach sexual maturity, nothing is known yet about males [21,22,23]. However, we hypothesise that a postdiapause period may also be necessary for males to reach sexual maturity, as irradiation during diapause could have stronger effects on sterility than irradiation after this period, which we know lasts three weeks in females.

Here, we report the first study testing the use of wild-harvested overwintering males of BMSB as an alternative option to mass rearing for the SIT. We evaluated the effects of irradiation on the fertility, longevity and mating behaviour of overwintering males of BMSB.

## 2. Materials and Methods

### 2.1. Insects and Rearing

During autumn of 2019 and 2020 (from the end of September until the end of November), 2500 and 3600 BMSB adults were, respectively, harvested in the field at Fondazione Edmund Mach (46°11′43″ N, 11°8′5″ E), San Michele all’Adige (Trento), Italy, using live traps [24]. The captured adults were reared in cloth cages (30 × 30 × 30 cm; BugDorm^®^, Taichung, Taiwan). Insects were provided with a diet consisting of tomatoes, green beans, carrots and apples ad libitum and wet cotton for a water supply. They were kept in a greenhouse with a natural photoperiod, where T = 18–15 °C and RH = 70%, as a form of preparation for overwintering until the end of November, when they were then transferred outside under a wooden shed. Cloth cages were set up with cardboard rolls as shelter, and a data logger (EL-USB-2, Lascar Electronics, Whiteparish, UK) was inserted into the wooden shed.

Insects were maintained in overwintering conditions for about two months, in accordance with the recommendations by Taylor et al. [22] that suggest a minimum of seven weeks before breaking diapause; the insects were then moved to a climatic chamber with standard conditions (L:D = 16:8, T = 25 °C, RH = 70%). However, to reduce possible negative effects on longevity due to thermal shock, BMSB adults were kept for three days at 18 °C before being moved to 25 °C. Afterwards, the cardboard rolls were removed, and the males and females were placed in separate cages to prevent them from mating before these experiments began. Then, we provided insects with the same diet mentioned above.

Considering the high mortality of individuals during overwintering, we chose to irradiate the insects at the end of the diapause. 

### 2.2. Irradiation 

Irradiation took place at the Radiation Oncology Department of Santa Chiara Hospital (Trento, Italy), using an alternative technique that involves the use of the Elekta Precise Sli linear accelerator (LINAC), from Elekta AB (Stockholm, Sweden), with high-energy photons (energy used: 6 MV) instead of the conventional method, based on Co-60, used in previous studies on BMSBs [18,19,20] and other hemipteran pests [25,26]. The decision to use a hospital LINAC (standard for treatment of oncological diseases) was dictated by the need to have an irradiator near the insect-collection site to reduce the time required to irradiate and maintain insect quality by limiting the stress associated with transport to an irradiator.

For accurate dose delivery, photons must pass through a material with a homogeneous density. To obtain this condition, we used water-equivalent materials (see irradiation details below). At the same time, we kept the insects in Petri dishes (diameter = 90 mm) containing wet, absorbent paper that reduced the presence of air inside; to achieve this, a phase of cold-induced torpor was deemed necessary. To chill the insects, they were placed in a plastic container (Ø = 133 mm; V= of 1180 mL) equipped with a cap with fine organza mesh and kept in a freezer at −20 °C for two minutes; then, the insects were transferred in a series of Petri dishes secured with parafilm. Each Petri dish contained an average of 15 individuals. The number of males irradiated for each treatment was in a range of 30–35 in January and February of 2020 and 60–62 in February of 2021. 

The Petri dishes containing the insects arranged for the irradiation were transported to the laboratories of the Santa Chiara Hospital in a polystyrene box. The temperature inside the box was regulated with an ice tablet and registered with a data logger; the values ranged between 5 °C and 10 °C. Once the insects were brought to the linear accelerator, four Petri dishes at a time were inserted in a plexiglass panel, measuring 30 × 30 × 2 cm, positioned between two layers, each consisting of five 30 × 30 × 1 cm sheets of a water-equivalent plastic material, PWW RW3 Slab Phantom^®^ (Freiburg, Germany), which simulates the density of water. The 4 mm space between the Petri dishes and the plexiglass was filled with four 1 mm-thick plexiglass disks. The entire structure was then placed under the LINAC. The irradiation was performed with two fields in the isocentric technique, one anterior and one posterior, with dimensions of 30 × 30 cm, so as to uniformly irradiate the entire system; the dose rate used was 250 cGy/min. Meanwhile, for a duration equal to the mean irradiation time (about five minutes), the Petri dishes containing the control insects with no irradiation (0 Gy, were kept outside the polystyrene box, in a room next to the irradiator, with the same room-temperature parameters. After irradiation, the insects were placed in cloth cages (24.5 × 24.5 × 24.5 cm; BugDorm^®^, Taichung, Taiwan), transported to the climatic chamber and fed with the same diet mentioned above until the formation of crosses for these experiments. The entire transport and irradiation procedure was performed within one hour.

### 2.3. Male Dose Response, Year 1

The first dose-response experiment was conducted in 2020, irradiating BMSB overwintering males at 16 and 24 Gy during two different physiological conditions: immediately after induced interruption of diapause (Group 1) and three weeks later (Group 2).

In particular, the data collected in terms of sterility were compared between Group 1 and Group 2 to understand if one of the two conditions were more suitable to induce stable sterility with irradiation. 

The males of Group 1 were irradiated during their dormancy (23 January 2020) and then fed for about three weeks in standard postdiapause conditions (L:D = 16:8, T = 25 °C, RH = 70%) until the females were ready for mating.

Before irradiation, the males of Group 2 were transferred into postdiapause conditions (23 January 2020), fed for three weeks and then irradiated (13 February 2020) and crossed with sexually reproductive females (14 February 2020).

A total of two treatments were tested for each group, plus the control. All crosses were carried out using overwintering males and females, as below.

Group 1:

(a) 0 Gy male × 0 Gy female (zero-dose control);
(b) 16 Gy male × 0 Gy female;
(c) 24 Gy male × 0 Gy female.

Group 2:

(a) 0 Gy male × 0 Gy female (zero-dose control);
(b) 16 Gy male × 0 Gy female;
(c) 24 Gy male × 0 Gy female.

A minimum of twenty replicates were performed for each treatment. Crosses were accomplished by confining each pair in a transparent plastic container (Ø = 133 mm and V = 1180 mL) equipped with a cap with fine organza mesh and a paper-towel sheet folded into four as oviposition substrates. 

Each pair was fed with two green beans and one cherry tomato; a 3 cm diameter plastic cup, in which was inserted a wet cotton, was utilised as a water source ad libitum. The diet and substrate were refreshed twice a week. 

BMSB pairs were checked twice a week to record the survival rate and oviposition. The collected egg masses were transferred into 90 mm-diameter Petri dishes and kept under the same climatic conditions to evaluate the hatching rate. Eggs that did not hatch were still checked for several weeks until they collapsed or blackened.

The oviposition experiment was concluded after three weeks (6 March 2020).

### 2.4. Male Dose Response, Year 2

During 2021, a second dose-response experiment was carried out using the same approach as for Group 2. BMSB males were transferred (12 January 2021) for 3 weeks in postdiapause conditions before being irradiated (2 February 2021) with X-rays at 16, 24, 32 and 40 Gy. The last two dosages were added with the aim to reach complete sterility.

Since it was the first time that we irradiated insects with this technique and several manipulations were required in preparation for irradiation, including transport to the irradiator, we decided to evaluate the effects of such variables on insects’ biology (longevity, fertility, fecundity) so that we could separate these effects from those due to the irradiation. Therefore, we introduced an additional control (0 Gy Cell) that was not prepared for irradiation and not transported to the irradiator but remained in the climatic chamber under the standard conditions used for all postdiapause insects (L:D = 16:8, T = 25 °C, RH = 70%). As in Experiment 1, the insects were mated after irradiation. Six treatments were carried out including the two controls:

(a) 0 Gy male × 0 Gy female (zero-dose Cell control);
(b) 0 Gy male × 0 Gy female (zero-dose control);
(c) 16 Gy male × 0 Gy female;
(d) 24 Gy male × 0 Gy female;
(e) 32 Gy male × 0 Gy female;
(f) 40 Gy male × 0 Gy female.

In the second year, the number of replicates (*n* = 40) per treatment was doubled from the previous year to obtain more-robust data. In addition, before starting of this experiment, twenty overwintering BMSB females were dissected to record the physiological statuses of the spermathecae according to Nielsen et al. [21], which were then compared with the data of fifteen one-week-old mated females with the expectation that spermathecal width (proximal part) would be significantly greater in mated than in overwintering females, confirming the unmated status of the latter. 

Parameters such as fertility of males, fecundity (expressed as the number of eggs laid by each ovipositing female) and longevity of both males and females were compared. The oviposition experiment was concluded with the death of the females. Likewise, their longevity was recorded until the death of all of the insects. 

### 2.5. Cumulative Mortality in F1

In order to detect the potential presence of inherited sterility, the survival of the F1 generation derived from the second experiment (male dose response, year 2) was also recorded at various development stages, from nymphs to adults, for 0 Gy, 32 Gy and 40 Gy to evaluate possible cumulative mortality effects due to irradiation. 

For each treatment, twenty-five first instars of BMSB were individually isolated in a transparent plastic container (Ø = 133 mm and V = 1180 mL) equipped with a cap with fine organza mesh. 

The insects were fed with a green bean, a cherry tomato and a shelled peanut, while a wet cotton ball (Ø ≈ 10 mm) was provided as a water source. 

The diet was refreshed twice a week. Insects were checked to record the survival rate and the stage (first to fifth nymphal instars or adult stage).

### 2.6. Mating-Behaviour Bioassays

Mating-behaviour bioassays were also performed to assess the mating performance of irradiated versus nonirradiated males in no-choice test conditions. The purpose was to evaluate possible differences in the durations and numbers of matings between irradiated males at the highest doses and nonirradiated insects.

A six-hour trial was carried out comparing irradiated overwintering adult males at 32 Gy and 40 Gy, with untreated (0 Gy) individuals. 

Crosses between different treatments were formed by confining one couple/treatment in a transparent plastic container (Ø = 133 mm and V = 1180 mL) equipped with a transparent cap. No food was provided during the test, except for water provided ad libitum with wet cotton in a small plastic cup. Bioassays were carried out at 25 °C, RH = 60% and an illuminance of ≈ 800 lx.

Three treatments were evaluated, with 12 replicates each:

(a) 0 Gy male × 0 Gy female (zero-dose control);
(b) 32 Gy male × 0 Gy female;
(c) 40 Gy male × 0 Gy female;

The number of matings and the duration of each mating were recorded based on direct observation. 

### 2.7. Statistical Methods

During the first year, differences in terms of fertility, measured in the egg-hatching percentage as a function of male irradiation dose, were evaluated among treatments (0, 16 and 24 Gy) and groups (1 and 2) using a generalised linear model with “quasibinomial” distribution.

In the second year, other parameters were evaluated. A Shapiro–Wilk test was performed to assess normality, and Levene’s test for homogeneity of variance was also performed to assess homoscedasticity. Because the parametric assumptions failed for all datasets, we adopted a nonparametric approach for data analysis:

(a) The mated status was ascertained by comparing the spermathecal widths of overwintering versus mated one-week-old females. A one-tailed Welch *t*-test [27], which considered the two means equal, was performed to test the null hypothesis.
(b) The fecundity of the females was compared using a two-sample Brown–Mood median test [28] followed by a contingency table (2 × 6) χ^2^. The number of egg masses produced per female and the number of eggs per egg mass were compared with the Kruskal–Wallis test.
(c) The fertility of males and the longevities of both males and females for the irradiated (16, 24, 32 and 40 Gy) and two unirradiated control groups (0 Gy Cell and 0 Gy) were compared through a Kruskal–Wallis test. In cases of significance, a Mann–Whitney pairwise test with the Bonferroni correction was then performed as a post hoc test.
(d) To evaluate possible cumulative mortality effects in the F1 generation among the two highest dosages of male irradiation (32 and 40 Gy) and a 0 Gy control, a χ^2^ test in a contingency table (2 × 3) was conducted for each stage (nymph from second to fifth instar and adult). Ryan’s test for multiple comparisons of proportions [29] was performed as a post hoc test.
(e) The numbers of matings, the mating durations and the total mating times were compared across treatments with the Kruskal–Wallis test, which was followed by the Mann–Whitney pairwise test with the Bonferroni correction as a post hoc test in cases of significance.

The differences between Group 1 and Group 2 in the first-year dose-response experiment were obtained in R [30] through the application of a generalised linear model (glm) with a quasibinomial distribution with the formula “H1 ~ Group * Radiation”, using the “lme4” package [31], where H1 is the egg-hatching percentage. 

The following tests were performed to analyse the second-year data using Past [32] (version: 4.12b): the Shapiro–Wilk test, Levene’s test, the χ^2^ test, the Kruskal–Wallis test and the Mann–Whitney pairwise test. 

## 3. Results

### 3.1. Male Dose Response, Year 1

**Figure 1:**
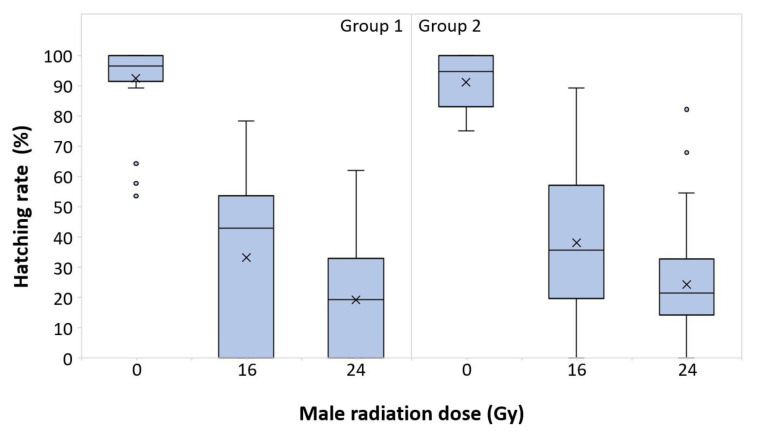
Hatching rates of all of the batches of eggs from crosses of irradiated (16 and 24 Gy) or untreated males (0 Gy), mated with virgin females of *Halyomorpha halys*, for Group 1 (**left**) and Group 2 (**right**). The upper whisker extends to the highest data value within the upper limit (Q3 + 1.5 (Q3 − Q1)); the boxplots represent the interquartile range, with a horizontal bar as the median, and the lower whisker extends to the lowest value within the lower limit (Q1 − 1.5 (Q3 − Q1)). The “×” symbol represents the mean value; the dots represent outliers.

The fertility (median percentage of hatching rate) was higher in the controls (96.49% for Group 1 and 94.64% for Group 2), decreasing when males were irradiated at 16 Gy (42.86% for Group 1 and 35.71% for Group 2) and declining up to 19.25% and 21.42% (respectively, for Group 1 and Group 2) at 24 Gy (Figure 1). The model performed did not display a significant difference in the effects of irradiation between the two groups nor in the interactions between groups and the male radiation dose in terms of hatching rate. On the contrary, significant differences in terms of fertility were found between the irradiated (16 and 24 Gy) and nonirradiated males (0 Gy) (Table 1).

The Group 2 protocol was selected as the most suitable, considering the high postdiapause mortality and the need to know precisely how many insects were available for experimental purposes.

### 3.2. Male Dose Response, Year 2

**Figure 2:**
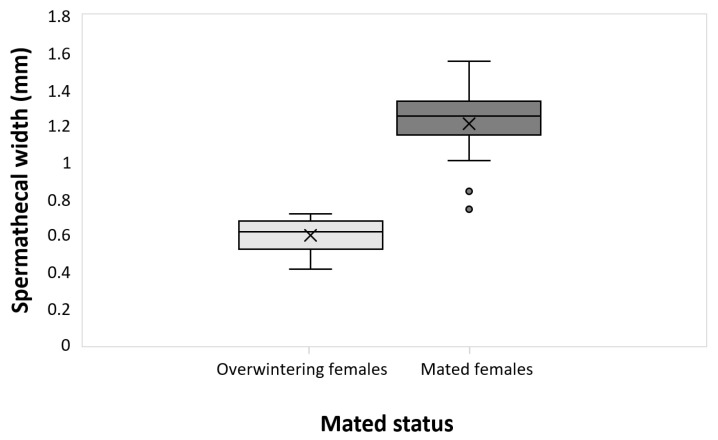
The mated status was verified as a function of spermathecal width by comparing the data between overwintering females and mated one-week-old females. The upper whisker extends to the highest data value within the upper limit (Q3 + 1.5 (Q3 − Q1)); the boxplots represent the interquartile range, with a horizontal bar as the median, and the lower whisker extends to the lowest value within the lower limit (Q1 − 1.5 (Q3 − Q1)). The “×” symbol represents the mean value; the dots represent outliers.

The mortality of the overwintering adults recorded in about two months of diapause was 59% for both males and females. The mortality of the remaining survivors in the following three weeks was 15% for males and 20% for females. As a result, 560 males and 600 females were available for experiments. The mated status of the overwintering females, verified with spermathecal width, was significantly different if compared with that of one-week-old mated females (t = −10.3; df = 17; *p* = 5.05 × 10^−9^), confirming the unmated status at the end of the diapause and consequently the suitability of overwintering females for these experiments (Figure 2). 

No significant differences were recorded between the groups in fecundity (eggs produced per female), number of egg masses per female or number of eggs per egg mass (Table 2).

**Figure 3:**
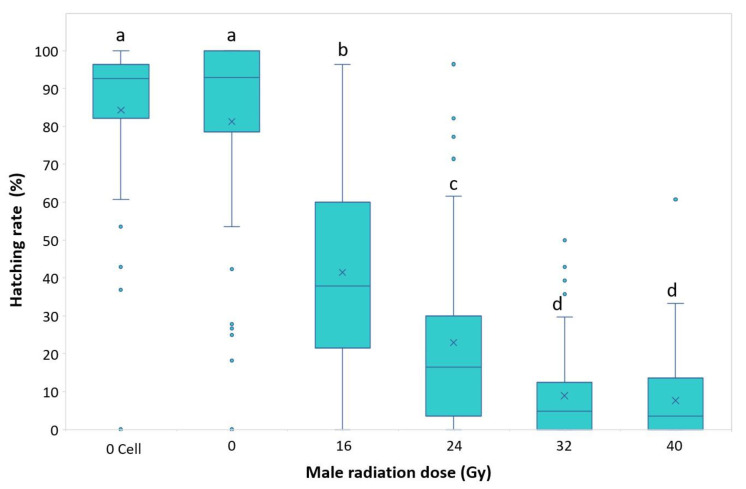
Hatching rates of all of the batches of eggs from crosses of irradiated (16, 24, 32 or 40 Gy) or untreated males (0 Gy Cell and 0 Gy) mated with virgin females of *Halyomorpha halys.* Letters (a–d) indicate differences among groups. The upper whisker extends to the highest data value within the upper limit (Q3 + 1.5 (Q3 − Q1)); the boxplots represent the interquartile range, with a horizontal bar as the median, and the lower whisker extends to the lowest value within the lower limit (Q1 − 1.5 (Q3 − Q1)). The “×” symbol represents the mean value; the dots represent outliers.

In terms of fertility (median), there was a significant difference among the treatments (Kruskal–Wallis test: H = 264.4; df = 447; *p* = 1.42 × 10^−55^). The results showed a declining trend from the two controls, 0 Gy Cell and 0 Gy, which had the identical value of 92.86%, through the irradiation dosages. The hatching rates for the doses of 16 and 24 Gy were, respectively, 37.93% and 16.39%, while they were 4.76% and 3.57%, respectively, for 32 and 40 Gy, which showed sterility of more than 95% (Figure 3).

**Figure 4:**
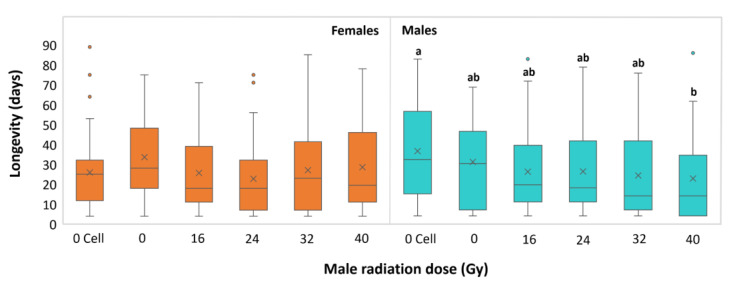
Longevities of untreated *Halyomorpha halys* females paired with irradiated males (**left**) and irradiated male adults (**right**), as a function of male irradiation dose. Letters (a, ab, b) indicate differences among groups. The upper whisker extends to the highest data value within the upper limit (Q3 + 1.5 (Q3 − Q1)); the boxplots represent the interquartile range, with a horizontal bar as the median, and the lower whisker extends to the lowest value within the lower limit (Q1 − 1.5 (Q3 − Q1)). The “×” symbol represents the mean value; the dots represent outliers.

In terms of longevity (Figure 4), no statistical difference was observed for females paired with irradiated (16, 24, 32 and 40 Gy) and unirradiated (0 Gy Cell and 0 Gy) males (Kruskal–Wallis test: H = 5. 234; df = 7; *p* = 0.28). Moreover, the mortality among the irradiated males was similar to that of both of the controls, except for the strongest dose (40 Gy), which was significantly different from the 0 Gy Cell group (Kruskal–Wallis test: H = 13.39; df = 239; *p* = 0.01). 

### 3.3. Cumulative Mortality in F1

**Figure 5:**
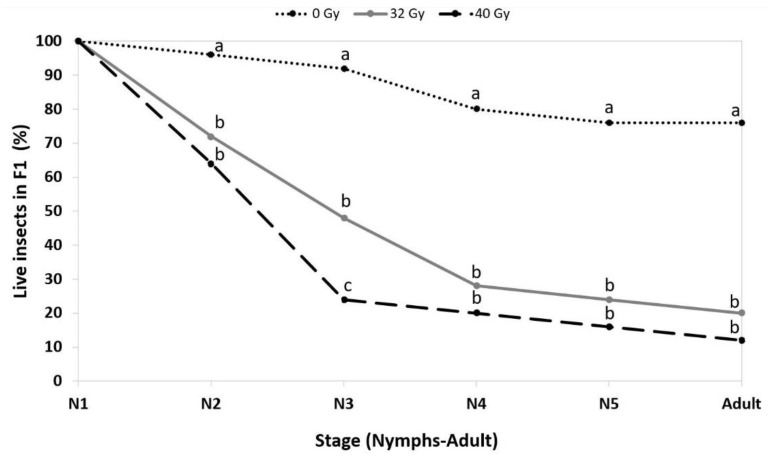
Percentage of insects alive at each stage in the F1 generation, considering the starting point (100% alive) of 25 first-instar insects. The different lines represent the treatments, and the letters (a–c) indicate the differences between the three treatments at each stage.

A clear difference in the F1-generation survival rate was recorded between the 0 Gy control and the two irradiation doses of 32 Gy and 40 Gy (Figure 5). 

The difference between the control of 0 Gy and the two radiation doses was significant starting from the second instar (χ^2^ = 7.91; df = 2; *p* = 0.019). As for the third instar (χ^2^ = 24; df = 2; *p* = 6.16× 10^−6^), all treatments differed from each other, whereas the fourth (χ^2^ = 21.7; df = 2; *p* = 1.95× 10^−5^) and fifth (χ^2^ = 24.74; df = 2; *p* = 4.26× 10^−6^) instars and also the adults (χ^2^ = 26.39; df = 2; *p* = 1.86× 10^−6^) showed significant differences only between the control of 0 Gy and the two irradiation doses.

To summarise, the percentages of F1 generation insects that reached the adult stage were 76% for 0 Gy and 20% and 12% for 32 Gy and 40 Gy, respectively.

### 3.4. Mating Behavioural Bioassays

In the six-hour mating trial, no statistically significant differences were found for the number of matings (Kruskal–Wallis test: H = 3.648; df = 34; *p* = 0.146) or the total mating time (Kruskal–Wallis test: H = 1.19; df = 34; *p* = 0.55). An increasing trend was observed in mating duration (mating time), with statistically significant differences between 40 Gy compared to 32 Gy and 0 Gy (Kruskal–Wallis test: H = 22.6; df = 162; *p* = 6 × 10^−11^) (Table 3).

## 4. Discussion

The success of an area-wide pest management programme that has the SIT as its strategic core relies on setting up a system to release an adequate number of sterile males able to compete with wild males for mating opportunities. Therefore, this work was focused on the selection of the correct irradiation dose, able to induce sperm sterility in irradiated males while at the same time maintaining the mating competitiveness of the sterile BMSB males with respect to the wild insects [19]. Given the difficulty in mass rearing of BMSBs, the possibility of using postdiapause overwintering males following large autumn mass trapping has been considered [19]. An irradiation technology that does not involve radioactive isotopes was also used, with the purpose of promoting the development of the SIT for future applications. In order to set up a suitable protocol to release competitive sterile males, it was crucial to consider the impact of diapause and postdiapause mortality of BMSBs. According to the data presented by Costi et al. [2], it seemed pointless to irradiate BMSB adults before their winter diapause. In fact, those authors reported that only 28% of adults were able to survive to the overwintering diapause when kept in outdoor conditions until spring, and only 50% of the surviving adults then reached reproduction. We thus focused our efforts to evaluate the performance of males that were irradiated to achieve sterility after surviving the postdiapause period. The results showed differences in hatching rates within the groups between the negative control, 0 Gy, and the two irradiation doses, 16 Gy and 24 Gy, but no differences between the groups. In the second dose-response experiment, performed during 2021, we chose the Group 2 protocol, considering it the most suitable, and applied irradiation only on the surviving males at the postdiapause mortality phase. 

The effects of irradiation on reproduction were different from the data presented by other authors who used gamma rays [18,19,20]. The rapid decline in fecundity observed by Welsh et al. [18] when a female mated with a male irradiated at 32 Gy or a higher dose was not recorded in our study. Furthermore, unlike what was reported by Welsh et al. [18], our hatching-rate data were higher, while they were lower than those reported by Suckling et al. [19] and more in line with those of Nguyen et al. [20]. Considering the same radiation doses, the observed differences could have depended on the different ages of the irradiated males [19]. 

Regarding effects on the longevity of irradiated males, our results are also different from the data already reported in the literature [18,20], where constant declines in longevity with increasing radiation doses were observed. Indeed, in the present study, only the males irradiated at 40 Gy showed significantly lower longevity than the control of 0 Gy Cell. Differently from what was reported by Welsh et al. [18], the longevity of the females was constant, independently from the irradiation dose of the male with whom a female was mating. The number of eggs laid per female was variable regardless of the male irradiation dose, as in the research of Nguyen et al. [20], unlike what has been observed in other works [18,19], which have reported declines related to higher radiation doses.

At the same time, while increasing the irradiation doses on males, we observed a negative impact on reproduction, with high levels of sterility at the highest dose. Moreover, the results of the cumulative mortality experiment showed that even if sperm sterility were not complete, the longevity and development of offspring were severely compromised (Figure 5). 

Even though most SIT programmes have been applied to target pests from Diptera and Lepidoptera [14,33], recent studies have shown the feasibility of including the SIT in area-wide control programmes against invasive alien pentatomids and even considering eradication strategies [19,25,26]. Concern about the suitability of implementing mass rearing facilities for the species belonging to pentatomids has led to new ideas and approaches based on mass harvesting, irradiation and release of large numbers of sterile males of the target pest species instead of multiplying them in a laboratory [19,26]. For this reason, recent studies have been addressed to develop a new live mass-trapping tool baited with the specific aggregation pheromone that enables collection of large numbers of BMSB adults during autumn [24]. The success of an SIT programme in area-wide IPM also depends on the quality of the insects to be released. In this context, it is essential to conduct studies on mating behaviour [34]. Often, poor performance of sterile males in terms of mating competitiveness has been wrongly attributed to side effects of irradiation [35,36]; on the contrary, the bottleneck is often due to the fact that prolonged captivity can induce drastic shifts, resulting in a clear increase in genotypes that are better adapted to indoor conditions rather than natural environments [26]. In the present study, the insects were wild-harvested, and thus their mating competitiveness would mainly have been influenced by irradiation. To assess possible side effects of irradiation on mating behaviour, mating times were recorded for the highest doses (32 and 40 Gy). The results showed that sterile wild-type BMSB males did not lose mating competitiveness when irradiated with the tested dose range. However, at 40 Gy, the mating time was significantly longer. To clarify these data, further studies should also consider sperm transmission and competition. Indeed, as modelled in a polyandrous species, in which it is uncertain whether a female would use the sperm of a single male, the effect of multiple matings can be assumed to depend on competition between sperm and between males [37,38]. Consequently, it will be crucial to understand whether the observed difference is related to sperm transmission and how females store sperm in the spermatheca.

The mating times recorded in our study are higher (12, 13 and 13.5 min for 0, 32 and 40 Gy, respectively) if compared to a previous study (10 min) [39] that described the mating behaviour of BMSBs. However, the experimental protocols were different: in our case, the insects were kept in separate pairs and were observed continuously, while in the previous study, the insects were all in the same container and data were collected every 10 min. Moreover, in the same work, it was observed that males required a 50–60 interval between successive coupling and that the average number of matings was 5.28 ± 0.15 in 24 h for both males and females [39]. In our study, the interval between matings was similar and the number of matings almost the same in comparing different treatments (6 for 0 Gy and 5 for 32 and 40 Gy), but the duration of the experiment was four times shorter (6 h versus 24 h). However, Kawada and Kitamura [39] observed that both males and females of BMSB can mate more than five times in a day, and these results are very relevant because those tests were performed immediately after sexual maturation.

Our team conceived mass field collection and exploitation of overwintering BMSB males [19], whose preliminary parameters for a possible practical application have been further defined in the present work. Indeed, it was necessary to test the feasibility of a programme involving efficient interruption of diapause that would not compromise the health or mating competitiveness of the insects, also considering the eventuality of transporting sterilised insects to another location (e.g., from one jurisdiction to another) and releasing them. In our case, the mortality recorded in about two months of diapause (59% for both males and females) and in the following three weeks (15% for males and 20% for females) was much lower than that recorded by Costi et al. [2], as the diapause was stopped long before spring. Furthermore, using suitable climatic chambers is possible to keep insects in optimal standard diapause conditions (at 9 °C) and optimise the survival of the insects until spring or, if necessary, successfully interrupt the diapause after only 7 weeks [22]. 

The 95% sterility rate achieved in this study at 32 Gy and 40 Gy is likely to be sufficient for suppression (if λ ≤ 20) according to the model constructed by Klassen and Creech [40]: *q* < 1/*λ* is applied when sterility is incomplete (where *q* is considered as a fraction of males that has remained fertile and λ is the population’s intrinsic rate of increase). Furthermore, to have effective suppression, the target pest species must be present in low numbers [33] and the ratio of “sterile males: fertile males” for BMSBs should be at least 5:1 [19]. 

Incomplete sterility is probably insufficient for eradication; nonetheless, if the SIT is used as a component of an IPM strategy, the possibility of having effective pest control in a limited area will increase. Indeed, use of the SIT as a component of area-wide pest management strategies, as demonstrated for Lepidoptera and Diptera, may effectively achieve both suppression and eradication of insect pests in many cases [33]. To incorporate BMSBs into an IPM programme, it is a primary factor to know the number of generations per year and the peak filial adult population, as that is the most damaging stage [41]. In invasion areas, both where BMSBs are univoltine, as in Switzerland [42], and where they are multivoltine, as in Southern Europe [2] and warmer United States areas [41], the only potential window of release for sterile, overwintering male populations is at the beginning of spring, when only the overwintering generation is present.

For BMSBs, the aspect concerning the damage caused by adults to crops remains critical; however, in an eradication programme, capture [24] and release of sterile BMSB males and any short-term and localised crop damage would not be considered as important as achieving removal long-term (risk–benefit analysis). Furthermore, it has been demonstrated on *Pistacia vera* (L.) (Sapindales: Anacardiaceae) that feeding damage caused by males is significantly lower than that caused by females [43]. 

Another important aspect in support of the concept of an area-wide IPM or eradication programme for management of BMSBs in its invasive range, with the involvement of the SIT, is the result of its synergism with classical biological control approaches [44,45]. 

Safety concerns related to implementation of radioactive sources for insect sterilization [46] can limit development of SIT programmes. Use of linear accelerators, which has already been explored in Diptera to induce sterility [47,48], can reduce irradiation costs and avoid the need for radioactive sources, thus eliminating the problems associated with safety, transport, handling and possible misuse of these materials [46,47,48]. 

High-energy photons produced by linear accelerators seem to be a valid alternative to other radiation sources and are suitable for some SIT programmes. In our study involving a 6 MV LINAC, the developed protocol allowed the achievement of a high level of sterility of overwintering males (over 95%) at only 32 Gy without compromising male mating behaviour (in no-choice conditions). Males’ longevity and fertility did not show significant differences between the two types of control, indicating that the abiotic stress related to the irradiation protocol was negligible up to 32 Gy. In addition to offering sterilisation of field-collected males as an alternative to expensive (and probably impossible) BMSB mass rearing, our results support use of medical linear accelerators that are present in hospital facilities, since these are more widely available compared to gamma sources, to promote SIT programmes. 

## Figures and Tables

**Table 1:** Results of the generalised linear model for hatching rates, showing the independent variables of the chosen model.

Coefficient	Estimate	Std. Error	t-Value	Pr(>|t|)
Intercept	−0.07863	0.02680	−2.934	0.00377 ** ^1^
Group 2	−0.01489	0.05438	−0.274	0.78447
Radiation, 16 Gy	−1.02482	0.12472	−8.217	3.39 × 10^−14^ *** ^1^
Radiation, 24 Gy	−1.57294	0.18732	−8.397	1.12 × 10^−14^ *** ^1^
Group 2; Radiation, 16 Gy	0.15456	0.15784	0.979	0.32874
Group 2; Radiation, 24 Gy	0.25251	0.22973	1.099	0.27311

**^1^** ** *p* < 0.01, *** *p* < 0.001.

**Table 2:** The percentage of egg-laying females was calculated on *n*1 (females alive after three days); in recording the first egg mass four days after the formation of the crosses, it was assumed that the females that died before the fourth day did not have enough time to lay eggs.

Male Dose (Gy)	*n*	*n1*	No. of Egg Masses	Total Eggs Collected	% Egg-Laying Females	Eggs/Egg Mass	Egg Masses/Egg-Laying Females	Eggs/Female
0 Cell ^2^	40	35	78	2052	60	26.31	3.75	56
0	40	36	79	2018	80.56	25.54	2.72	50
16	40	35	75	1954	57.14	26.05	3.45	41.5
24	40	32	46	1185	43.75	25.76	3.29	27.5
32	40	35	98	2513	62.86	25.64	4.45	95.5
40	40	33	71	1827	48.48	25.73	4.60	96
*p*						0.9 ^1^	0.09 ^1^	0.16 ^1^

^1^ The *p*-values were calculated with a 95% confidence interval. ^2^ The 0 Cell control is the negative control: insects that were not irradiated or transported to the irradiator.

**Table 3:** Numbers of matings, mating times and total mating times are shown in the table as medians. For the mating times, the letters “a” and “b” indicate the differences among the groups.

Male Dose (Gy)	*n*	No. of Matings	Mating Time (min)	Total Mating Time (min)
0	12	6	12 a	72
32	11	5	13 a	64
40	12	5	13.5 b	66
*p*		0.15 ^1^	5.997 × 10^−6 1^	0.55 ^1^

^1^ The *p*-values were calculated with a 95% confidence interval.

## Data Availability

The data presented in this study are available on request from the authors.
